# Ancient DNA reveals the timing and persistence of organellar genetic bottlenecks over 3,000 years of sunflower domestication and improvement

**DOI:** 10.1111/eva.12594

**Published:** 2018-02-13

**Authors:** Nathan Wales, Melis Akman, Ray H. B. Watson, Fátima Sánchez Barreiro, Bruce D. Smith, Kristen J. Gremillion, M. Thomas P. Gilbert, Benjamin K. Blackman

**Affiliations:** ^1^ Department of Plant and Microbial Biology University of California Berkeley CA USA; ^2^ Department of Biology University of Virginia Charlottesville VA USA; ^3^ Centre for GeoGenetics Natural History Museum of Denmark University of Copenhagen Copenhagen Denmark; ^4^ The Santa Fe Institute Santa Fe NM USA; ^5^ Department of Anthropology Ohio State University Columbus OH USA; ^6^ Norwegian University of Science and Technology University Museum Trondheim Norway

**Keywords:** ancient DNA, archaeobotany, domestication, genetic bottleneck, *Helianthus annuus*, paleogenomics, plant evolution, sunflower

## Abstract

Here, we report a comprehensive paleogenomic study of archaeological and ethnographic sunflower remains that provides significant new insights into the process of domestication of this important crop. DNA from both ancient and historic contexts yielded high proportions of endogenous DNA, and although archaeological DNA was found to be highly degraded, it still provided sufficient coverage to analyze genetic changes over time. Shotgun sequencing data from specimens from the Eden's Bluff archaeological site in Arkansas yielded organellar DNA sequence from specimens up to 3,100 years old. Their sequences match those of modern cultivated sunflowers and are consistent with an early domestication bottleneck in this species. Our findings also suggest that recent breeding of sunflowers has led to a loss of genetic diversity that was present only a century ago in Native American landraces. These breeding episodes also left a profound signature on the mitochondrial and plastid haplotypes in cultivars, as two types were intentionally introduced from other *Helianthus* species for crop improvement. These findings gained from ancient and historic sunflower specimens underscore how future in‐depth gene‐based analyses can advance our understanding of the pace and targets of selection during the domestication of sunflower and other crop species.

## 1 INTRODUCTION

Over the last 12,000 years, human populations in many different regions of the world independently domesticated local plant species by selecting for desirable traits, in many cases initiating a symbiotic partnership that formed the economic foundation of complex societies (Zeder, 2015). Researchers have identified over a dozen centers of plant domestication (Purugganan & Fuller, 2009), and gaining a refined understanding of the varied evolutionary trajectories that have led to the emergence of key crops requires investigating the cultivars and the archaeological context found in each of the world's independent centers of domestication. Eastern North America (ENA) presents a useful case to examine initial plant domestication and millennial‐scale changes in agriculture (Smith, 2011), in part because its archaeological record challenges the paradigm that domestication is an evolutionary strategy implemented when expanding human populations experience declining resource catchments (Smith, 2016). Starting around 4000 years before present (BP), a crop complex consisting of acorn/crookneck squash (*Cucurbita pepo* L. ssp. *ovifera* D.S. Decker), goosefoot (*Chenopodium berlandieri* Moq.), marshelder (*Iva annua* L.), and the common sunflower (*Helianthus annuus* L.) was grown by low‐level food‐producing societies inhabiting the watershed of the Mississippi River (Smith, 2006). Archaeobotanical remains from ENA sites exhibit telltale signs of the so‐called domestication syndrome (Hammer, 1984), a suite of traits that commonly distinguishes domesticates from their wild progenitors and that may include larger seeds and disruption of natural seed dispersal mechanisms. Of the four core species of the ENA crop complex, sunflower is particularly well suited for in‐depth domestication research thanks to the existence of rich archaeobotanical collections (Smith, 2014), a century of breeding experiments (Heiser, 1976; Škorić, 1992), and the development of many germplasm and genomic resources for genetic investigations (Badouin et al., 2017; Burke, Tang, Knapp, & Rieseberg, 2002; Kane et al., 2011; Rieseberg & Seiler, 1990; Wills & Burke, 2007).

Through human selection, the weedy *H. annuus* spp. *annuus* was transformed from a highly branching plant with numerous small disks, also known as heads or capitula, to *H. annuus* spp. *macrocarpus* (D.C.) Ckll., the cultivated sunflower, which is typically characterized by strong apical dominance and a single massive disk that can produce hundreds to thousands of achenes. Sunflowers served important nutritional, ceremonial, medicinal, cosmetic, and structural purposes in Native American cultures. For instance, an account from 1615 by French explorer Samuel de Champlain indicates that peoples of the Iroquois Confederacy of Nations in the Great Lakes region of North America cultivated sunflower, grinding and eating the seeds as well as processing them into oil used ceremonially for anointing the hair (Heiser, 1951). After roasting sunflower achenes in clay pots or reed baskets, the Mandan, Arikara, and Hidatsa peoples of the Missouri River basin would make sunflower flour or boil the achenes with maize, beans, and squash to make a porridge (Heiser, 1976). The Hopi people of the American Southwest were unique in extracting a dye from the deeply purple‐colored achenes of their landraces (Heiser, 1951, 1976).

**Figure 1:**
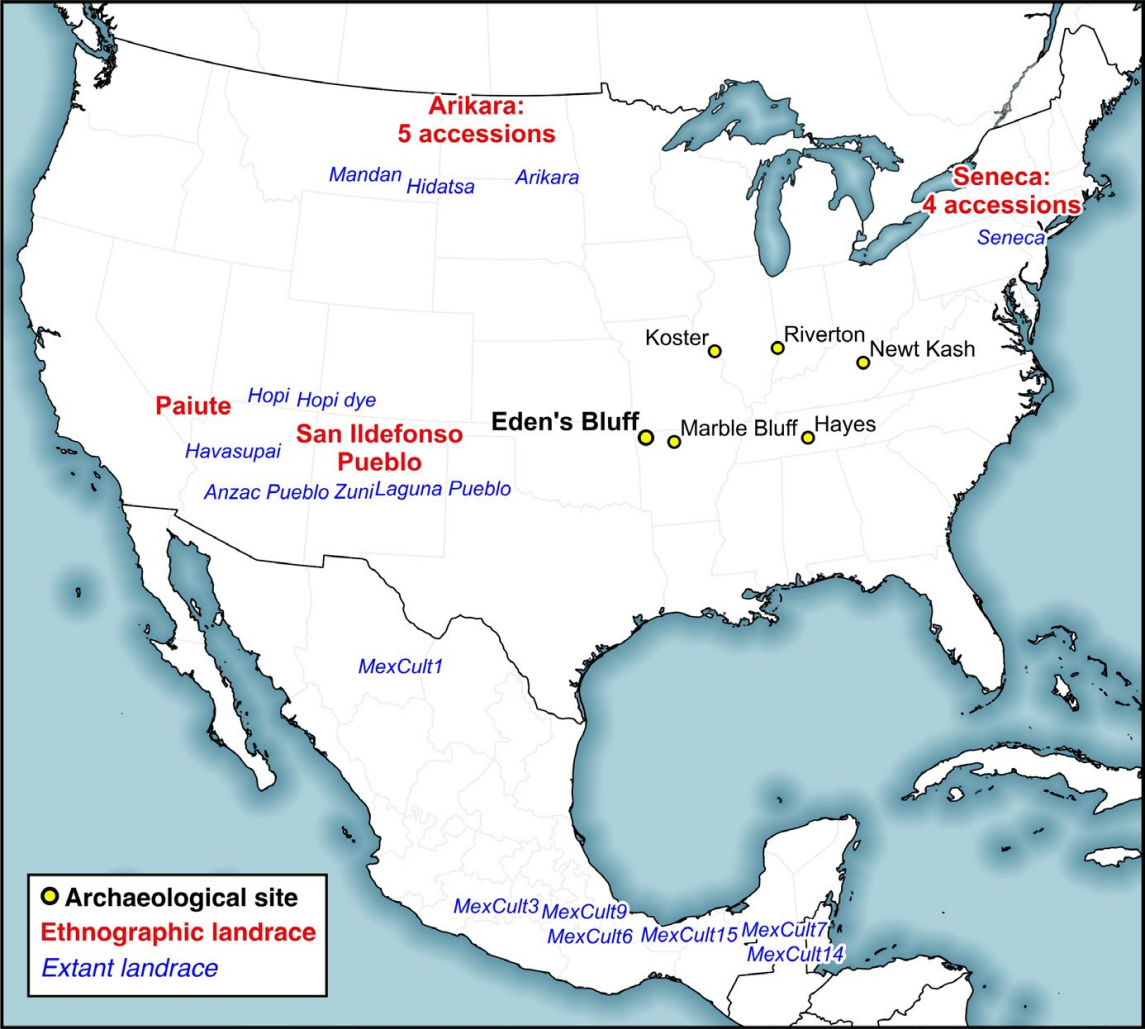
Map of sampling locations and archaeological sites. Ethnographic samples (and number of accessions sampled) are in red, and landraces are in blue. Archaeological sites with ancient sunflower material discussed in the text are marked by yellow circles. Eden's Bluff, the site from which all archaeological remains detailed in this article were sampled, is bolded

Archaeological sunflower remains have been excavated from dozens of ENA sites, enabling temporal and spatial investigations on the origins of sunflower domestication. The Koster site in Illinois yielded the oldest known sunflower remains, with two achenes and one kernel dating between 8500 and 5800 BP (Asch & Asch, 1985; Smith, 2014) (Figure 1). Based on their small size, these specimens likely reflect the collection of wild resources (Smith, 2014). The oldest evidence for sunflower cultivation comes from the Hayes site in central Tennessee, dating to 5034–4583 BP (95% confidence interval, CI) (Crites, 1993). Kernels from the site are larger than commonly observed in wild sunflowers, suggesting the initial steps of sunflower domestication were underway circa 4800 BP (Smith, 2014). Three other sites provide evidence of sunflower cultivation before 3000 BP (Figure 1): 3800 BP at the Riverton site in Illinois (Smith & Yarnell, 2009), 3300 BP at the Newt Kash Shelter in Kentucky (Smith, 2014), and 3050 BP at the Marble Bluff Shelter in Arkansas (Fritz, 1997).

Based on archaeological, morphological, and geographical data, Heiser (1951) concluded that sunflower was domesticated once in ENA, a hypothesis that has been supported by population genetics studies of modern elite‐bred cultivars, extant Native American landraces, and wild *H. annuus* populations. For instance, Rieseberg and Seiler (1990) demonstrated with isozymes and chloroplast markers that domesticated landraces share haplotypes with wild sunflowers from ENA and show a signature of a genetic bottleneck. Although archaeological remains putatively identified as sunflower, some dating to 4130 BP, were subsequently recovered from excavations in Mexico and raised the possibility of an independent domestication event (Lentz, Pohl, Pope, & Wyatt, 2001), population genetic studies that include extant Mexican wild and cultivated germplasm have only found evidence that extant cultivars derive from a single ENA domestication event. Wills and Burke (2006) showed that domesticated populations have one common and two rare chloroplast microsatellite marker haplotypes that cluster with wild ENA rather than wild Mexican sunflowers. Patterns of sequence variation at nuclear microsatellite markers and candidate domestication loci have likewise reinforced the conclusion that all extant landraces, whether collected in ENA or Mexico, descend from a single origin most likely occurring from ancestral wild populations in the eastern and central USA (Blackman et al., 2011; Harter et al., 2004).

Although archaeological and genetic data predominantly point to a single domestication event in ENA, there is much more to unearth about how sunflower domestication proceeded. It remains to be determined which traits were of primary interest to early farmers, whether sunflower domestication was rapid or protracted, and how proto‐domesticates responded to the new selection regime. Genetic characterization of archaeological plant remains with ancient DNA (aDNA) methodologies has the potential to answer these questions by providing windows into past temporal dynamics. Paleogenomic research has grown tremendously in the past decade due to the rapid development of high‐throughput sequencing technologies (Der Sarkissian et al., 2015), and the application of paleogenomic methods to archaeobotanical remains has been a particular success (Brown et al., 2015). For example, in reconstructing complete genomes of 6,000‐year‐old barley grains excavated in Israel, Mascher et al. (2016) determined the ancient samples were closely related to modern cultivars in the region and that the major steps of barley domestication were completed by this point in time. Similarly, Ramos‐Madrigal et al. (2016) and Vallebueno‐Estrada et al. (2016) characterized genomes of 5,000‐year‐old maize cobs from the Tehuacán Valley, but they instead found that many domestication‐related genes had the ancestral form rather than the derived maize form, suggesting a stepwise process of domestication. Although these paleogenomic studies indicate archaeological remains could be invaluable for understanding sunflower's domestication and ancient cultivation, different plant species have the potential to confound aDNA research through species‐ and tissue‐specific secondary compounds that interfere with DNA extraction and library preparation. To examine the paleogenomic potential of archaeobotanical sunflower remains, we screened a collection of archaeological and ethnographic specimens with a shotgun sequencing strategy. The sequencing data generated from these ancient and historic specimens were analyzed to determine variability in endogenous content, DNA damage, and sources of exogenous DNA. In addition, following precedents in mammalian aDNA projects (Dabney, Knapp, et al., 2013; Gilbert et al., 2007) and genome skimming of modern samples (Bock, Kane, Ebert, & Rieseberg, 2014; Straub et al., 2012), we leveraged the sequencing data to characterize variation in high copy number mitochondrial and plastid genomes, allowing us to investigate how these and other archaeological and historic specimens may enrich our understanding of the domestication process.

## 2 MATERIALS AND METHODS

### 2.1 Archaeological sunflower specimens

Although archaeobotanical remains are most often preserved by charring or carbonization, such materials are generally incompatible with paleogenomic analyses (Nistelberger, Smith, Wales, Star, & Boessenkool, 2016). Therefore, we only obtained and processed desiccated specimens for this study. We tested 15 sunflower disk fragments, one pericarp (seed coat), and one kernel, all of which originate from the Eden's Bluff archaeological site in northwestern Arkansas (Figures 1 and S1; Table 1). The specimens have been under the curation of the University of Arkansas Collections Facility (UARK) and the University of Michigan Museum of Archaeological Anthropology (UMMAA). Thirteen disks that were sufficiently intact to enable diameter measurements ranged in size from 35 to 110 mm (mean = 75.5) and were all larger in this dimension than disks of a well‐defined wild *H. annuus* population (Smith, 2014), indicating the archaeological disks represent plants cultivated by humans. Likewise, the dimensions of the archaeological pericarp (length = 9.1 mm) and kernel (length × width = 6.5 × 3.6 mm) are consistent with origin from domesticated sunflowers.

**Table 1:** Archaeological specimens. Accelerator mass spectrometry (AMS) dates are listed in calibrated years before present. Samples with sequencing depth of coverage (DoC) <4 for the plastome were excluded from the plastome analysis. See Figure S1 for images of most samples and Table S1 for additional sample, AMS, and sequencing information

Specimen	Tissue	calBP (95% CI)	Endogenous DNA	Plastome DoC
Eden‐1	Pericarp	Not dated	0.3%	0.8
Eden‐2	Disk fragment	915–795	11.9%	5.4
Eden‐3	Disk fragment	3168–3005	2.1%	3.2
Eden‐4	Disk fragment	Not dated	0.2%	0.3
Eden‐5	Disk fragment	1736–1574	14.1%	8.3
Eden‐6	Disk fragment	3163–2999	5.6%	7.2
Eden‐7	Disk fragment	1813–1622	8.6%	7.6
Eden‐8	Disk fragment	1817–1628	19.1%	4.1
Eden‐9	Disk fragment	1819–1633	48.3%	16.5
Eden‐10	Disk fragment	1873–1629	35.3%	5.6
Eden‐11	Disk fragment	1825–1618	31.1%	17.5
Eden‐12	Disk fragment	1868–1701	34.1%	24.0
Eden‐13	Disk fragment	1810–1571	55.6%	18.1
Eden‐14	Disk fragment	1877–1711	9.9%	8.3
Eden‐15	Disk fragment	1770–1559	25.7%	30.6
Eden‐16	Disk fragment	1819–1639	35.6%	14.7
Eden‐17	Kernel	Not dated	0.1%	1.1

Eden's Bluff (state site ID: 3BE6) was excavated in 1932 and 1934 as a part of expeditions led by the University of Arkansas focused on the so‐called Ozark Bluff‐Dweller sites, as coined by Harrington (Harrington, 1924a, 1924b, 1960). These sites are renowned for their preservation of organic remains, including desiccated plant tissues (Fritz, 1986; Gilmore, 1931). Native Americans likely used the rockshelters and caves specifically because their dry conditions were well suited for long‐term food storage and, despite the name, are unlikely to have served as seasonal dwellings (Brown, 1984). The chronology of the Ozark Bluff‐Dweller sites is not fully understood, due to the limited number of radiocarbon dates (Davis, 1967). As part of her rigorous archaeobotanical analyses, Fritz (1986) acquired dates from 15 sites and determined occupations occurred throughout the period from ca. 3000–500 BP. Because their stratigraphic context may have experienced disturbance from humans, rodents, or other causes, we submitted 14 of the 17 samples for direct accelerator mass spectrometry (AMS) radiocarbon dating at the University of Arizona AMS facility (Table 1; Figure S2). All AMS dates from this and other reports were calibrated to calendar years before present (calBP) using OxCal v4.3.2 (Bronk Ramsey, 2009) and the IntCal13 (Reimer et al., 2013) calibration curve.

### 2.2 Ethnographic landrace achenes

Eleven accessions of sunflower landraces were acquired from ethnological collections at the National Museum of the American Indian (NMAI) and UMMAA (Table 2, Figure 1). These specimens consist of achenes sourced from Native Americans and via various intermediaries in the first half of the twentieth century by Gilmore (1919) and Heiser (1951). At the time, Heiser (1951, p. 441) lamented that “few aboriginal strains of the cultivated sunflower are still in existence, and… it is likely that the few remaining ones will disappear unless steps are taken to preserve them.” While his efforts propagated many sunflower landrace lineages, some of the achenes he attempted to grow were not viable, including seed originating from the Six Nations reserve in Ontario. Thus, these ethnographic achenes offer a unique opportunity to investigate genetic relationships of putatively extinct landraces to living sunflower lineages.

**Table 2:** Ethnographic achenes from Native American sunflower landraces. Three Seneca achenes are reported to have been collected in North Dakota (indicated with an asterisk); however, oral traditions and written records indicate these landraces originated from the traditional lands of the Seneca people near Lake Ontario

Specimen	Repository	Location	Year	Collector
Arikara 122976	NMAI	Fort Berthold Reservation, North Dakota	1923	M. R. Gilmore
Arikara 126306	NMAI	North Dakota	1924	M. R. Gilmore
Arikara 14042‐874	UMMAA	Bismarck, North Dakota	1932	George F. Will
Arikara broad 12999‐682	UMMAA	Bismarck, North Dakota	N/A	George F. Will
Arikara/Mandan 13747	UMMAA	Dakotas	1933	M. R. Gilmore
Paiute 141856	NMAI	Moapa River Reservation, Nevada	1920s	M. R. Harrington
San Ildefonso Pueblo 13597‐747	UMMAA	San Ildefonso Pueblo, New Mexico	N/A	Jose Aguilav
Seneca 137749	NMAI	Allegany Reservation, New York	1925	W. Wildshut
Seneca purple 12996‐682	UMMAA	Bismarck, North Dakota*	1931	George F. Will
Seneca purple 12998‐682	UMMAA	Bismarck, North Dakota*	1931	George F. Will
Seneca striped 12997‐682	UMMAA	Bismarck, North Dakota*	1931	George F. Will

NMAI, National Museum of the American Indian; UMMAA, University of Michigan Museum of Archaeological Anthropology.

### 2.3 DNA extraction and sequencing

Archaeological specimens were processed at a dedicated paleogenomics laboratory at the University of Copenhagen. The laboratory meets the standards for aDNA research (Cooper & Poinar, 2000; Gilbert, Bandelt, Hofreiter, & Barnes, 2005), such as being physically separated from modern DNA and post‐PCR laboratories, being outfitted with air filtration and nightly UV irradiation equipment, and requiring researchers to wear coveralls to minimize contamination. DNA was extracted using a method that has been shown to work well on a range of species and tissue types (Wales, Andersen, Cappellini, Ávila‐Arcos, & Gilbert, 2014). In brief, tissue samples were collected with disposable forceps and scalpels, placed in PowerBead tubes (MO BIO 13117‐50), and pulverized by shaking at 4 m/s for 30 s in a FastPrep‐24 homogenizer (MP Biomedicals). The resulting tissue powder was incubated overnight in a digestion buffer (10 mM Tris‐HCl, 10 nM NaCl, 2% w/v SDS, 5 mM CaCl_2_, 2.5 mM EDTA, 40 mM DTT, and 10% proteinase K solution), and then extracted using two rounds of phenol and one round of chloroform. To minimize the effect of co‐extracted compounds and pigments, the recovered DNA was purified in a Qiagen MinElute column using optimizations to retain highly fragmented DNA (Dabney, Knapp, et al., 2013). Four extraction blanks were processed with samples to monitor potential sources of contamination. The extracted DNA, including that from the extraction blanks, was converted to Illumina‐compatible libraries using a blunt‐ended adapter ligation approach and optimizations to retain short molecules (Wales et al., 2015). Before indexing PCR, the libraries were tested by quantitative PCR (qPCR) to estimate the appropriate number of cycles to avoid overamplification. qPCR was conducted with a SYBR Green assay as described by Wales et al. (2015), using AmpliTaq Gold (Applied Biosystems, Foster City, CA), primers IS7 and IS8 (Meyer & Kircher, 2010), and a Roche LightCycler 480 Real‐time PCR System. Libraries were amplified with AmpliTaq Gold for 10–18 cycles (Table S1) using a P7 indexing oligo with a 6‐bp sample‐specific barcode to enable multiplex sequencing (Meyer & Kircher, 2010). Libraries were pooled and shotgun‐sequenced on six whole or partial lanes of an Illumina HiSeq 2500 in single‐read mode with 81 or 94 sequencing cycles (Table S1).

The 11 ethnographic samples were deemed to be relatively well preserved and thus to pose a potential contamination risk to archaeological samples. Therefore, the achenes were extracted in sterilized laminar flow hood in a pre‐PCR modern DNA laboratory at the University of Copenhagen where sunflowers had not been previously tested. Achenes were frozen in liquid nitrogen and fragmented with a sterile pestle. DNA was extracted with a Qiagen Plant Mini kit following the manufacturer's protocol except that the 65°C incubation was conducted for 2 hr. Many specimens exhibited high‐molecular‐weight DNA on an agarose gel, so DNA was sheared with a Diagenode Bioruptor using an appropriate number of sonication cycles for each sample (Table S1). One accession (Seneca_striped_12997‐682) was processed twice, using a whole achene and an individual kernel. DNA was converted to Illumina libraries following the same protocol used for the archaeological samples and sequenced on one lane of an Illumina HiSeq 2500 in single‐read mode with 81 sequencing cycles.

### 2.4 Sequencing data processing

Raw sequencing reads were processed using Paleomix 1.2.12 (Schubert et al., 2014), a bioinformatic pipeline developed for aDNA datasets. The recommended parameters for paleogenomic datasets were utilized, including removing adapter sequences with AdapterRemoval 2 (Schubert, Lindgreen, & Orlando, 2016), mapping of reads with BWA aln with the seed disabled (Li & Durbin, 2009), removal of duplicate reads with Picard Tools (http://broadinstitute.github.io/picard), realignment around indels with GATK 3.7 (McKenna et al., 2010), and rescaling of base qualities due to aDNA damage with mapDamage2.0 (Jónsson, Ginolhac, Schubert, Johnson, & Orlando, 2013). Reads were mapped against the entire sunflower XRQ draft genome (Badouin et al., 2017), including unplaced contigs, the plastid genome, and the mitochondrial genome. We report endogenous DNA content based on all mapped reads, regardless of mapping quality, because high content of long terminal repeat retrotransposons in the sunflower genome (74.7% of the genome, Badouin et al., 2017) cause many endogenous reads to map to multiple loci. As we observed potential erroneous insertions of the organellar genomes in the nuclear assembly, reads were also separately mapped to the plastid genome, mitochondrial genome, and the nuclear genome without unplaced contigs; these alignments were only used for organellar genome and library complexity analyses.

To place the archaeological and ethnographic samples in context, publicly available sequencing data from 79 modern cultivars, 20 landraces, 27 wild *H. annuus* individuals, and 47 individuals of 4 other annual *Helianthus* species were downloaded from the NCBI sequence read archive (SRA) (Table S2). Because they were sequenced with deep coverage, we subsampled and analyzed 30 million paired reads for each modern cultivar to reduce computational time. The entire datasets were used for the other samples. Raw data were processed in the Paleomix pipeline as discussed above, except that the mapDamage rescaling of base qualities was omitted. To minimize potential biases arising from differences in sequencing strategies, such as higher theoretical mapping scores from paired‐end than single‐read data, the paired‐end modern data were treated as though it was single‐read data by trimming and mapping read mates separately.

### 2.5 Metagenomic analysis of archaeological and ethnographic samples

To characterize non‐sunflower sources of DNA isolated from archaeological and ethnographic specimens, 10,000 randomly selected trimmed, unmapped reads were compared against the NCBI nucleotide collection (nr/nt) database using the BLASTn algorithm (Altschul, Gish, Miller, Myers, & Lipman, 1990). MEGAN6 (Huson, Mitra, Ruscheweyh, Weber, & Schuster, 2011) was used to taxonomically group BLASTn results with LCA parameters: Min Score = 10, Max Expected = 10, Min Percent Identity = 0.0, Top Percent = 0.0001, Min Support Percent = 0.0, Min Support = 1, Min Complexity 0.0, LCA algorithm = weighted, Percent to cover = 80, and ReadAssignment Mode = readCount. MEGAN6 was used to perform a principal coordinate analysis (PCoA) of Bray–Curtis distances of taxonomic grouping at the genus level, excluding all assignments to Viridiplantae.

### 2.6 Organellar DNA analysis

Reads mapping to the plastome (plastid or chloroplast genome) or to the mitochondrial genome were processed with GATK 3.7 (McKenna et al., 2010) HaplotypeCaller and GenotypeGVCFs tools to identify polymorphic sites. Polymorphisms were filtered with GATK according to recommended parameters for depth, mapping quality, strand biases: QD < 2.0, MQ < 30.0, FS > 60.0, SOR > 3.0, MQRankSum < −12.5, and ReadPosRankSum < −8.0. The sites were further filtered with VCFtools (Danecek et al., 2011) to exclude indels and retain SNPs with a quality score >1,000. Archaeological samples with <4× average coverage of the plastome genome were excluded from the analysis. SNPs were analyzed in R 3.3.1 (R Core Team, 2013) using the Pegas (Paradis, 2010) package to identify haplotypes, and then, haplotype relationships were visualized in popart (Leigh & Bryant, 2015) using a minimum spanning network (Bandelt, Forster, & Röhl, 1999). For construction of the haplotype networks, a total of 701 and 413 polymorphic sites were used for the plastome and mitochondrial genome, respectively. One of the oldest samples (Eden‐3) together with three other archaeological samples (Eden‐1, Eden‐4, and Eden‐17) did not satisfy our filtering parameters and thus were not included in haplotype network construction.

### 2.7 Organellar nucleotide diversity analysis

Nucleotide diversity (pi) per each polymorphic site was computed using VCFtools (Danecek et al., 2011) allowing for haploid genomes (haploid switch). For each group, mean nucleotide diversity was calculated by taking average nucleotide diversity of all the sites used in haplotype network construction for chloroplast or mitochondria. Landrace diversity metrics were calculated after excluding MexCult7 and MexCult14 because those samples were collected in local markets in Chiapas/Mexico and are likely modern cultivars as inferred from the haplotype networks.

## 3 RESULTS

### 3.1 Chronology

AMS radiocarbon dating of the archaeobotanical remains demonstrated the specimens originate from three distinct time points: 3100, 1700, and 850 calBP (Figure S2). Eden‐3 and Eden‐6 are the oldest samples, producing nearly identical AMS dates (Table S1), and thereby provide strong evidence that Eden's Bluff should be added to the short list of archaeological sites with sunflower cultivation before 3000 BP. Eleven AMS dates fall near 1700 calBP, all of which overlap at a 95% CI from 1736 to 1711 calBP. Thus, the majority of the samples may be derived from a single occupational phase; however, these specimens are recorded as being excavated from multiple contexts, suggesting that some specimens may have been deposited decades or even a few centuries apart. Eden‐2 produced the youngest date at ca. 850 calBP (Table 1). While this young disk is an outlier in the chronology of our other AMS dates, Fritz (1986) found similar dates for maize excavated from Eden's Bluff, supporting the inference that this sample belongs to a more recent occupation.

### 3.2 Shotgun sequencing and endogenous content

**Figure 2:**
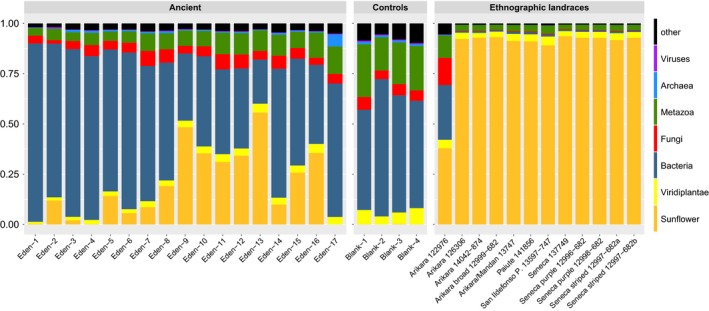
DNA content of ancient and ethnographic landrace samples and extraction controls. Percentage of total reads mapping to the sunflower genome and relative proportion of unmapped reads assigned to kingdom‐level taxa based on a random sampling of 10,000 unmapped reads

We generated 4.1–30.6 million raw sequence reads for the archaeological specimens (mean = 12.4 M), 0.41–0.68 M reads for the four controls (mean = 0.53 M), and 15.9–35.8 M reads for the ethnographic achenes (mean = 23.2 M) by Illumina sequencing. The archaeological specimens exhibit endogenous DNA contents ranging from 0.17% to 55.66% (mean = 21.1%, median = 16.6%), with both achenes and one disk yielding <1% endogenous DNA (Table 1, Figure 2). For 11 of the 12 ethnographic specimens, 89.1%–93.6% of DNA mapped against the reference genome. In the remaining ethnographic sample, Arikara 122976, only 37.9% of the reads were endogenous (see exogenous DNA below). Aside from one sample with low endogenous content (Eden‐17), nuclear DNA PCR duplicate levels were low for the Illumina libraries on the archaeological (mean 3.06%, median = 0.32%) and ethnographic specimens (mean = 1.42%) (Table S1). These low levels indicate that the libraries contain a great amount of untapped complexity and could be deeply sequenced to recover large portions of the nuclear genome.

### 3.3 DNA degradation

Consistent with the findings from previous paleogenomic studies, DNA recovered from the archaeological sunflowers was highly fragmented and displayed varying levels of chemical damage (Figure S3). The mean read length of endogenous nuclear DNA for archaeological samples ranged from 41.9 to 62.1 bp, with an overall mean of 52.6 bp (Table S1). Cytosine deamination is the principal form of damage observed in aDNA studies (Dabney, Meyer, & Pääbo, 2013), and in circumstances where contamination from modern sources is possible, especially hominin research, damage patterns can be used to discern ancient and modern sequences (Jónsson et al., 2013). During the life of a cell, cytosine residues can spontaneously convert to uracil, but they are fixed with cellular repair mechanisms. After death, these uracil residues accumulate, primarily in single‐stranded overhangs, and due to the activity of polymerases used in DNA library preparations, apparent C‐to‐T and G‐to‐A transitions are observed at the 5′ and 3′ ends of sequencing reads. This damage can be visualized as ski‐jump style plots (Figure S3), with steeper slopes indicating more damage. In addition, the δS parameter calculated by mapDamage provides a probability of cytosine deamination in single‐stranded contexts (Table S1). Our samples produced δS values ranging from 0.165 to 0.999 (mean = 0.605). As anticipated from well‐preserved, relatively recent specimens, the ethnographic samples exhibit low levels of damage (δS range = 0.018–0.056, mean = 0.035). The ethnographic DNA is also less fragmented than that of the archaeological samples. Although Arikara_14042‐874 is an outlier with an average length of 59.3 bp, library fragments frequently exceeded the length of the number of sequencing cycles (mean read length = 77.4 bp, sequencing length = 81 bp), and this mean is artificially reduced as high‐molecular‐weight DNA was extracted from many ethnographic samples and needed to be fragmented by sonication prior to library construction.

### 3.4 Exogenous DNA

Metagenomic analysis of unmapped reads revealed a complex mixture of DNA in archaeological and control samples (Figure 2). The chief contaminant across all archaeological samples is bacteria (up to 85%) with Actinobacteria primarily differentiating archaeological samples from ethnographic samples (PC1, Figure S4). The extraction controls are also dominated by bacteria, and taxa such as Proteobacteria, Actinobacteria, and Firmicutes are consistent with species commonly observed as laboratory reagent contaminants (Salter et al., 2014). Fungi and metazoans also make up a substantial proportion of archaeological contaminants, contributing as much as 30% of read content in several samples.

Taxonomic assignment of unmapped reads at the genus or species level can help identify problematic individual samples and highlight methodological or biological factors that require further examination. For instance, the majority of unmapped reads in ethnographic samples are broadly assigned to the Viridiplantae, but most of these have top BLAST hits to the *H. annuus* genome. These reads may not have mapped to the sunflower genome due to sequence divergence from the reference genome and/or because the BLASTn algorithm as applied was more tolerant of polymorphism than BWA. Ethnographic samples also have on average >3 times more unmapped reads assigned to chordates (18.5% compared to 5.4% in archaeological samples) and animal parasites such as Platyhelminthes and Apicomplexa (5.9% and 1.9% compared to 2.0% and 0.5% respectively in archaeological samples). Eden‐1 and Eden‐2 are differentiated from other archaeological samples (PC2 in Figure S4) by high counts of Gammaproteobacteria (specifically the *Pseudomonas stutzeri* group in Eden‐1 and *Pseudomonas putida* group and Enterobacteriales in Eden‐2). One ethnographic sample, Arikara_122976, more closely resembles archaeological samples with lower endogenous sunflower DNA content (37.9% compared to the ethnographic average 87.6%) and a more substantial fraction of sequences originating bacterial, fungal, and metazoan contaminants. While Arikara_122976 groups with archaeological samples in the PCoA analysis (Figure S4), it contains nearly twice as many unmapped reads assigned to fungi, with most assigned to the Sordariomycetes, as any other ethnographic sample (Figure 2).

### 3.5 Plastome analysis

**Figure 3:**
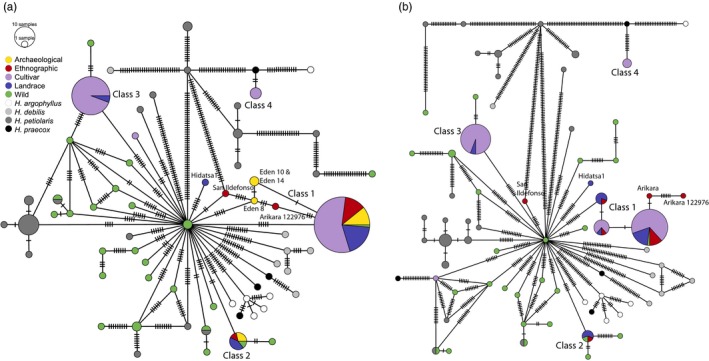
Plastome haplotype networks constructed with wild, cultivated, landrace, ethnographic, and archaeological sunflowers (a), and plastome haplotype network constructed without the archaeological sunflowers (b). The size of the circles corresponds to number of individuals present, and the number of polymorphic sites between individual haplotypes is indicated by tick marks. Haplotype classes for each sample are included in Table S3. Class 1 is a core domestication haplotype and is composed of wild *Helianthus annuus*, archaeological specimens, ethnographic samples, extant landraces, and modern cultivars. Class 2 also represents a haplotype that entered the domestication process thousands of years ago; however, it is not observed in cultivars. Class 3 consists of R‐type elite cultivars used in hybrid breeding, and was presumably introduced into domesticated germplasm from *H. petiolaris* in the 20th century; as discussed in the text, we suspect two Mexican landraces in Class 3 may originate from misidentified cultivars. Class 4 consists exclusively of elite cultivars, and was likely introduced from crop wild relatives, putatively *H. argophyllus*, during recent breeding for resistance to pathogens and diseases

We constructed two haplotype networks, one including and one excluding the archaeological samples (Figure 3). Exclusion of the archaeological samples provides for greater haplotype resolution of the ethnographic samples, as the greater level of missing data in the archaeological data reduces the number of polymorphic sites informative for network construction.

The cultivated sunflower sequences—whether from archaeological or ethnographic remains, extant landraces, or modern cultivars—sort into few haplotype clusters that we have denoted as Classes 1 through 4 relative to the much greater diversity observed in wild *Helianthus* sequences, which are nearly all unique (Figure 3; Table S3). All Eden's Bluff archaeological specimens dating to ~1700 calBP fall in Class 1 and share the same or similar haplotypes as many ENA, southwestern, and Mexican landraces; several ethnographic samples; and the majority of modern cultivars (Figure 3a). Although Eden‐8, Eden‐10, and Eden‐14 have distinct haplotypes, they are only one or two substitutions removed from the predominant Class 1 haplotype. Many more substitutions must be inferred to support the reticulate lineages connecting their sequences to the distinct Arikara or San Ildefonso haplotypes or to any other wild *H. annuus* plastome sequence, and the more resolved structure of Class 1 in the haplotype network excluding the archaeological samples suggests those alternative connections are highly unlikely to reflect the true history of descent (Figure 3b).

The two other Eden's Bluff samples for which sufficient sequence was recovered for plastome analysis—Eden‐2 (850 calBP) and Eden‐6 (3100 calBP)—cohere with the third most common haplotype class, Class 2. This group also includes three Mexican landraces collected from Nahua farmers (MexCult3, MexCult6, and MexCult9), an ethnographic Seneca sample, and a wild *H. annuus* individual from Texas; however, no modern cultivars share this sequence. It is important to note the existence of Class 2 haplotypes in extant landraces would not be known without David Lentz and Robert Bye's painstaking survey in Mexico (Lentz, Pohl, Alvarado, Tarighat, & Bye, 2008). In contrast to Class 2, Class 3, the second most common haplotype class, has a membership consisting nearly entirely of R‐type modern cultivars, which are lines carrying a nuclear restorer allele for the cytoplasmic male sterility system used for hybrid sunflower breeding. Two putative Mexican landraces (MexCult7 and MexCult14) also carry the Class 3 plastome sequence, raising the possibility they are actually elite‐bred material. The Class 4 haplotype sequence shared by three modern cultivars (BRS‐1, HA‐R2, and IR) is most similar to sequences obtained from annual *Helianthus* species other than *H. annuus*, likely reflecting a history of introgression as part of a recent breeding program. Finally, the Hidatsa landrace has a unique haplotype compared to other samples analyzed, consistent with the findings of a previous study of sunflower sequence diversity using chloroplast microsatellite markers (Wills & Burke, 2006).

### 3.6 Mitochondrial genome analysis

**Figure 4:**
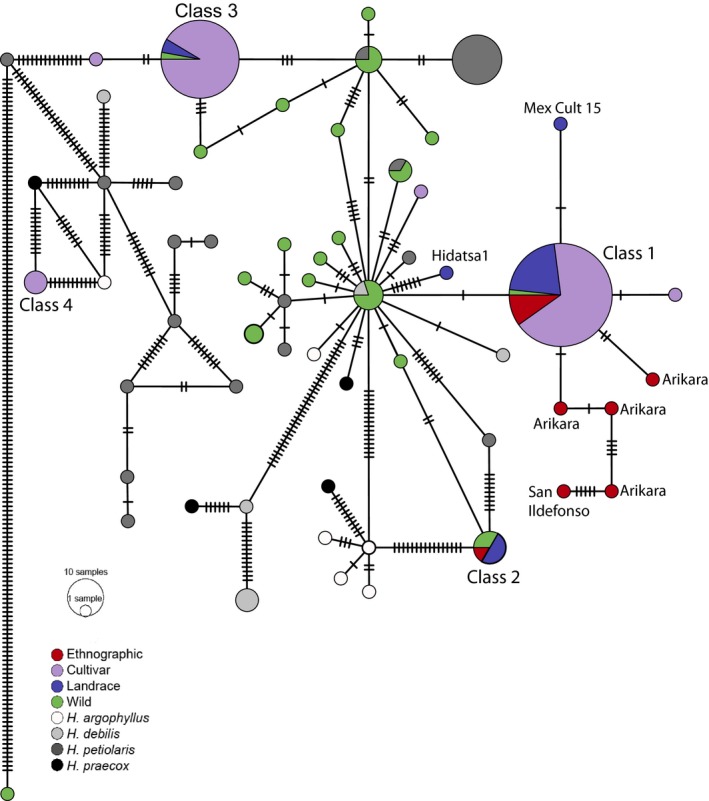
Mitochondrial haplotype network constructed with wild, cultivated, landrace and ethnographic sunflowers. The size of the circles corresponds to number of individuals present, and the number of polymorphic sites between individual haplotypes is indicated by tick marks. Haplotype classes for each sample are included in Table S3. Class 1 is composed of individuals sharing the same haplotype and also those that diverge by only one or two polymorphic sites. Due to uniparental inheritance of organelles, the mitochondrial classes contain the same individuals as the plastome classes. See Figure 3 for information on the domestication haplotypes (Classes 1 and 2) and those introduced to modern cultivars during 20th‐century breeding (Classes 3 and 4)

When archaeological sequences are excluded, the haplotype network constructed for mitochondria is very similar to the plastome network. Four major cultivated haplotype classes emerge with nearly the same memberships, and thus, we use parallel nomenclature (Figure 4, Table S3). One key difference is that the San Ildefonso ethnographic sample is more similar to the Class 1 cultivated haplotypes than to any other cultivated or wild mitochondrial sequence. Inclusion of mitochondrial sequences from the Eden's Bluff samples in network construction analysis led to poorly resolved, highly reticulate networks. In contrast to the observed plastome sequences, each of the mitochondrial haplotypes from these archaeological samples contained many apparent private mutations causing each sample to appear unique. We suspect these patterns are artifactual, likely reflecting spurious SNPs originating from short exogenous DNA sequence fragments that align to highly conserved regions or, alternatively, SNPs that originate from nuclear inserts of mitochondrial DNA (Hazkani‐Covo, Zeller, & Martin, 2010; Thalmann, Hebler, Poinar, Pääbo, & Vigilant, 2004).

### 3.7 Nucleotide diversity

The average pairwise nucleotide diversity (pi) of all groups of domesticated sunflower samples is reduced relative to wild *H. annuus*, consistent with a genetic bottleneck during domestication (Table 3). This reduction is comparable for both organellar genomes. For instance, there is a 68% and 72% reduction in diversity in ethnographic samples compared to wild *H.  annuus* in chloroplast and mitochondria, respectively. Within domesticated types, modern cultivars have higher sequence diversity relative to the ethnographic samples and landraces. However, this likely reflects the recent introgression of wild haplotypes by modern breeding, as cultivars and landraces show lower diversity as compared to the ethnographic samples when only the diversity within the major haplotype classes also present in the Eden's Bluff samples (Class 1 and 2) is considered (Table 3). We report a value for pi for the archaeological samples but note that this metric is best suited for analyses of contemporaneous individuals and that diversity within a single site is generally expected to be lower than diversity present in the broader geographical sampling represented by the sequences from wild, ethnographic, or modern cultivated material.

**Table 3:** Nucleotide diversity (pi) for wild, archaeological, ethnographic, landrace, and modern cultivated sunflowers. It is important to note that the archaeological specimens were excavated from one site and are therefore not wholly comparable to population‐level measures of pi for the other sunflower groups. Given that Class 3 and 4 haplotypes were likely introduced to domesticated lines during recent breeding, a separate calculation of pi for modern cultivars with Class 1 and 2 haplotypes is provided

Sunflower group	Nucleotide diversity in plastome	Nucleotide diversity in mitochondria
Wild	0.0403	0.0458
Archaeological	0.0099	N/A
Ethnographic	0.0127	0.0126
Landrace	0.0125	0.0073
Class 1 and 2 landrace	0.0094	0.0050
Modern cultivar	0.0285	0.0235
Class 1 and 2 modern cultivar	0.0091	0.0084

## 4 DISCUSSION

### 4.1 Sunflower archaeological remains yield quality endogenous DNA

While aDNA studies have revealed important insights into the pace of selection during domestication in some plants (e.g., Mascher et al., 2016; Ramos‐Madrigal et al., 2016; Vallebueno‐Estrada et al., 2016), recovery of degraded DNA from most crops is not routine, and this project represents the first exploration of how paleogenomic testing of archaeological sunflower remains can be used to understand its unique domestication history. Through paired AMS dating and paleogenomic testing of archaeological specimens from the Eden's Bluff site in Arkansas, we find that many desiccated remains dating back as far as 3100 BP can be valuable sources of DNA. Some specimens yield more than 50% sunflower DNA, although a seemingly random subset of specimens yield levels of endogenous DNA (<1%) essentially incompatible for state‐of‐the‐art paleogenomic techniques, such as targeted enrichment of genetic loci of interest (Carpenter et al., 2013). Still, 13 of the 17 specimens yielded >5% endogenous DNA and are therefore well suited for in‐depth analysis of nuclear targets that can be defined from genomic and transcriptomic studies of extant sunflower germplasm.

We suspect the exogenous DNA content obtained from our samples originates from at least four sources: organisms that inhabited the disks and achenes during the life of the plant, such as pathogens; organisms that consumed metabolites, proteins, and other biomolecules in the tissue after the death of the individual; environmental DNA transferred from the archaeological sediment; and modern DNA contamination from excavation, curation, and genetic testing. While it is difficult to distinguish these potential sources, the sequencing of extraction controls provides a means to identify cross‐contamination of samples and pervasive DNA in laboratory reagents (Salter et al., 2014).

We observed that DNA degradation patterns are variable in archaeological sunflower, both in terms of DNA fragment length and the frequency of chemical damage, even within one relatively tight time interval. For example, the two oldest specimens (Eden‐3 and Eden‐6) yielded effectively identical AMS dates of ca. 3100 calBP. However, compared to Eden‐6, Eden‐3 has slightly shorter endogenous DNA (difference of means = 5.8 bp) and higher levels of cytosine deamination (δS of 0.999 vs. 0.673). Similarly, the youngest sample from the collection, Eden‐2, dates to 850 calBP and has DNA that is nearly as short (mean fragment length of 62.1 bp) and as damaged as Eden‐9 (mean fragment length of 59.7 bp), which is twice as old. Thus, fragmentation and damage profiles do not necessarily follow straightforward, time‐dependent degradation patterns, perhaps reflecting variability in how different remains were treated prior to deposition (e.g., intentional desiccation or heating in antiquity). Together, these findings indicate that multiple samples from the same site and stratigraphic layer ought to be initially tested by low‐depth shotgun sequencing to identify promising candidates for in‐depth genetic analysis.

### 4.2 Organellar haplotype networks recapitulate anticipated patterns for extant taxa

Organellar genomes in most plants exhibit uniparental inheritance (Sato & Sato, 2013). Therefore, a one‐to‐one association of plastid haplotypes with mitochondrial haplotypes is often expected (Mogensen, 1996), and indeed, we observe such a tight correspondence between our defined organellar haplotype classes (Table S3). Because the plastid and mitochondrial genomes are nonrecombining, it can be possible to use organellar loci as markers for taxonomic identification, as is performed with DNA barcoding studies (Avise et al., 1987; CBOL Plant Working Group et al., 2009). Yet, the organellar genomes of the five annual *Helianthus* species we have sampled do not resolve into mutually exclusive clusters in either haplotype network. Such patterns are consistent with previous findings demonstrating substantial gene flow between *Helianthus* species and/or incomplete lineage sorting (Sambatti, Strasburg, Ortiz‐Barrientos, Baack, & Rieseberg, 2012; Whitney et al., 2015). For instance, Bock et al. (2014) observed a similar lack of taxonomic structure in the organellar genomes of perennial *Helianthus* species, suggesting this is common throughout the genus.

Most modern cultivars carry one of two distinct haplotypes (the most common Class 1 sequence or Class 3), and these assort into inbred line classes developed to facilitate hybrid production. Elite‐bred sunflower lines are classifiable into two types: male “R‐lines” and female “B‐lines,” the latter being derived from open‐pollinated varieties (OPV) (Korell, Mösges, & Friedt, 1992). The Class 1 chloroplast and mitochondrial haplotypes observed in extant germplasm are predominant among B‐lines and OPVs as well as most extant landraces, suggesting that this cluster contains the few organellar sequences that passed through the domestication and improvement bottlenecks. The 33 modern cultivars in our survey that carry the Class 3 haplotype are all R‐lines, which carry a mitochondrial mutation (PET‐1) introgressed from *H. petiolaris* Nutt. that causes male sterility as well as a nuclear restorer allele (*Rf*) for this mutation (Balk & Leaver, 2001). As expected based on this breeding history, the mitochondrial haplotype of Class 3 groups closely with sequences present in *H. petiolaris* (Figure 4). Because only *Rf* is required to restore fertility in hybrid crop breeding, we do find two R‐type cultivars, RHA‐418 and RHA‐401, in the Class 1 haplotype cluster. The shared breeding history of RHA cultivars likely also explains the divergence between Class 1 and Class 3's plastome haplotypes. Although the plastome haplotype of Class 3 does not have clear affinity for any of the obtained *H. petiolaris* sequences, it is possible that more similar *H. petiolaris* plastome haplotypes were not included among the individuals sampled. Two putative Mexican landraces (MexCult7 and MexCult14) share the Class 3 plastome and mitochondrial haplotypes. Unlike other Mexican landraces, which were obtained directly from native farmers, these domesticates were obtained from an open marketplace in Chiapas, Mexico (D. Lentz, personal communication; Blackman et al., 2011). Thus, the possibility that they may in fact be seeds derived from modern R‐type sunflower lines is plausible and merits rigorous examination in whole genome analyses.

Another case of deliberate introgression is observed for the third, less common, cultivar haplotype: Class 4. The Class 4 organellar haplotype is most similar not to other *H. annuus* sequences but instead to sequences from other annual *Helianthus* species. This observation is consistent with published breeding information for at least two of the three Class 4 carrying cultivars. BRS‐1 and HA‐R2 are derived from the OPV Argentinian Impira INTA cultivar, which is a hybrid of *H. argophyllus* and *H. annuus* var Saratov Permgamino, and were selected for disease‐resistant traits (Bertero de Romano & Norberto Vázquez, 2003).

Overall then, while three organellar genome types predominate in modern cultivated germplasm, these very distinct Class 3 and Class 4 sequences are not shared with landraces, ethnographic, or archaeological samples and have largely entered cultivated *H. annuus* through recent, deliberate introgression of genetic material from other wild *H. *species. The history of directed breeding of domesticated sunflower lines with crop wild relatives strongly suggests Class 3 was introduced from *H. petolaris* during the establishment of the hybrid crop agricultural system (Seiler, Qi, & Marek, 2017). Class 4 was likely also introduced during crop improvement, potentially from *H. argophyllus*, the sunflower species which has been most frequently crossed with domesticated lines to impart disease and parasite resistance (Seiler & Fredrick Marek, 2011). Indeed, it is perhaps surprising that additional non‐*H. annuus* haplotypes were not more commonly observed, as breeders have introduced allelic variation for novel traits (e.g., resistance against a range of pathogens) by prolific and repeated introgression of genetic material from other *Helianthus* species. *H. annuus* has reportedly been crossed with every annual species and 14 perennial species in the genus (Kaya, 2014). Our finding of only two introgressed haplotypes, one of which was deliberately selected for, likely reflects that *H. annuus* has predominantly served as the recurrent maternal parent during sunflower improvement.

### 4.3 Ethnographic and archaeological organellar sequences reveal lost diversity and raise new hypotheses

Although low‐depth shotgun sequencing data from ancient samples like those which we report here generally do not enable population‐level characterization of nuclear genes of interest, patterns of variation in organellar genomes can be assessed because these DNA sources are found in many copies per cell, increasing their chance of recovery (Hofreiter, Serre, Poinar, Kuch, & Paabo, 2001). Furthermore, analyses of nonrecombining loci from archaeological samples can lead to important insights about the phylogeography and demography of domestication, as demonstrated by aDNA studies of pigs (Larson et al., 2007), cattle (Beja‐Pereira et al., 2006), and bottle gourds (Kistler et al., 2014).

The sequences that we recovered from archaeological and ethnographic sunflower samples provide new information about the extent and timing of the bottlenecks in genetic diversity accompanying domestication and improvement that have previously been inferred from extant sunflower sequences (Baute, Kane, Grassa, Lai, & Rieseberg, 2015; Liu & Burke, 2006). Although nearly every wild *H. annuus* individual carries a unique plastid haplotype, the archaeological and ethnographic samples assort into just two haplotype clusters. Notably, the most common haplotype among both modern and historical domesticated forms (Class 1) was present at Eden's Bluff at least 1,700 years ago, as were two additional closely related but distinct haplotypes not represented in any extant germplasm (Figure 3a). Given these sequences are separated by fewer substitutions from the major domesticate haplotype than from any wild haplotype, we infer these are more likely to represent de novo evolution following a domestication bottleneck than retention of standing variation from the wild ancestor. Likewise, we observe several more unique Class 1 haplotypes that are satellites of the major haplotype among the ethnographic samples, and the Class 2 haplotype observed in the oldest Eden's Bluff sample and several Native American landraces are completely absent from elite‐bred cultivars. Together, these findings suggest that all domesticated sunflowers likely coalesce to very few maternal lineages present early in the domestication process. Given that the archaeological samples analyzed in this study are from a single site and might not fully reflect the genetic diversity present in the earliest phases of domestication, aDNA analysis of additional archaeological samples will be important for affirming these findings.

In addition, our results confirm Heiser's lament; Native American landraces once harbored genetic diversity now absent from modern germplasm. Absence of the Class 2 haplotype and the unique ethnographic Class 1 haplotypes in elite cultivars likely reflects genetic bottlenecks imposed during 20th‐century improvement programs and by the subsequent rise of the lines produced to agricultural dominance throughout North America (Heiser, 1976; Škorić, 1992). The loss of diversity in extant landraces relative to historic samples also provides a caution and an opportunity for conducting genome scans for domestication genes. By including nuclear DNA recovered from ethnographic specimens, it may be possible to distinguish between genes that experienced selective sweeps as a consequence of the domestication process versus changes in sequence diversity that score similarly by population genetic metrics due to the recent loss of landrace germplasm. The sole modern wild *H. annuus* sample carrying a Class 1 haplotype is also instructive in this regard. Given the frequency at which domesticated and wild sunflowers interbreed (Arias & Rieseberg, 1994; Linder, Taha, Rieseberg, Seiler, & Snow, 1998) and that this individual was collected in California, well outside the proposed ENA domestication center, we expect it acquired the Class 1 haplotype by gene flow from contemporary domesticates. Thus, this finding highlights the importance of vetting putatively wild sunflower individuals for signals of admixture prior to inclusion in genomic scans for selective sweeps.

The archaeological and ethnographic haplotype sequences we have recovered are also consistent with a single center of sunflower domestication located in ENA. Both Class 1 and Class 2 haplotypes were present at Eden's Bluff before 1700 calBP, and both classes are also observed in historic and extant landraces. The presence of the distinct Class 2 haplotype at Eden's Bluff at 3100 calBP and in three Mexican landrace accessions but also a Seneca ethnographic sample does introduce some ambiguity because the pattern fails to be fully diagnostic for a single ENA origin versus an additional second center of domestication of sunflower in Mexico, as suggested by Lentz et al. (2008, 2001). Nonetheless, the single domestication hypothesis remains the most compelling conclusion for multiple reasons. First, the three Class 2 Mexican landraces were all collected from indigenous Nahua farmers in the state of Guerrero (Blackman et al., 2011) who spoke only Nahuatl and yet did not know the Nahuatl word for sunflower (D. Lentz, personal communication). Thus, it is possible that these landraces were introduced to this region of Mexico more recently than the early domestication period. Second, two wild individuals from the central United States (northern Texas) carry the Class 2 haplotype. Thus, if these do not represent admixed genotypes and if further sequencing of Mexican wild populations fails to yield the Class 2 sequence, then a Mexican origin can be excluded. Finally and most persuasively, multilocus nuclear genotype data and candidate domestication gene sequences from these three Mexican landraces demonstrate they are more closely related genetically to extant landraces and wild populations from ENA than to wild populations in Mexico (Blackman et al., 2011).

Because we have obtained aDNA sequence for archaeological samples excavated at the same site but that date to three separate time periods, we can compare the Eden's Bluff samples not only to wild germplasm from the modern era but also to each other. In doing so, we observe a pattern of sequence turnover. The samples dated to the earliest and latest time points (3100 calBP and 850 calBP) both carry the Class 2 haplotype, but the many samples dated to the intermediate time interval (1700 calBP) possess the Class 1 haplotype exclusively. This pattern suggests that multiple different domesticated lineages of sunflowers were maintained in the region for millennia and might reflect differential cultivation of these proto‐landraces across time. It is interesting to note that these time points generally correspond to major prehistoric cultural periods in the Ozarks and across North America, namely the Late Archaic, Woodland, and Mississippian periods (Sabo & Early, 1990). Despite these potential links to cultural changes, it must be emphasized that we have tested a limited number of samples and many samples dating to 1700 calBP could originate from one depositional episode from a small group of farmers. Thus, there is a chance that both Class 1 and Class 2 would be observed throughout the stratigraphic sequence at Eden's Bluff if more samples were characterized. Nonetheless, this intriguing pattern of turnover makes clear the powerful potential of aDNA to raise and to investigate new hypotheses about domestication and cultural history that have left no footprint in the genomes of extant germplasm. Future studies of nuclear genome sequence from these samples and aDNA from other remains obtained over time in this region are sure to reveal further insights into the temporal and spatial dynamics with which early sunflower landraces arose and spread to other regions.

## 5 CONCLUSIONS AND FUTURE DIRECTIONS

In summary, we have shown that recovery of ancient and historic DNA from archaeological and ethnographic sunflower specimens is feasible and that desiccated specimens frequently contain high levels of endogenous DNA. At present, shotgun sequencing data allow us to infer the relationships between ancient and modern samples for organellar loci. In tandem with sequencing data from modern accessions, we have gained new perspectives on the persistence of plastid lineages for thousands of years under cultivation and the loss of genetic diversity during recent improvement. We recognize these loci track the maternal lineage and do not document the full domestication history of the sunflower, and our future studies where we obtain greater depth of coverage for many loci in the nuclear genomes of ancient and historic specimens will allow us to address more nuanced questions about the pace of domestication and specific targets of selection. Fortuitously, numerous desiccated archaeological specimens have been excavated from dozens of sites in the Ozarks and other parts of ENA (Fritz, 1986; Gilmore, 1931; Smith, 2014), thereby providing the means to identify genetic changes over millennia. Most of the specimens were excavated from rockshelters from 1920 to 1930 (Davis, 1967; Harrington, 1924a, 1924b, 1960), but some of these sites, including Eden's Bluff, have since been inundated by the construction of dams in the mid‐20th century or otherwise degraded (Fritz, 1986). Thus, these curated specimens offer an otherwise unachievable prehistoric perspective on sunflower domestication. Candidate targets of selection during domestication have been reported in several studies (Baute et al., 2015; Blackman, Strasburg, Raduski, Michaels, & Rieseberg, 2010; Blackman et al., 2011; Chapman, Mandel, & Burke, 2013; Chapman et al., 2008), and identifying more should be accelerated thanks to expanding genomic resources being generated by the International Consortium for Sunflower Genomic Resources (Badouin et al., 2017; Kane et al., 2011). Thus, we anticipate paleogenomic characterization of archaeological and ethnographic sunflower tissues will soon have tremendous potential to resolve long‐standing questions about the demographic and functional history of domestication for this important oilseed crop.

## DATA ARCHIVING

Data for this study are available at the NCBI SRA: BioProject ID PRJNA422624.
