# Supplementary figures and images for: Ancient DNA reveals the timing and persistence of organellar genetic bottlenecks over 3,000 years of sunflower domestication and improvement
Source: Evol Appl. 2018 Feb 13;12(1):38–53. doi: 10.1111/eva.12594 (PMC6304678; doi:10.1111/eva.12594)

Eden-2

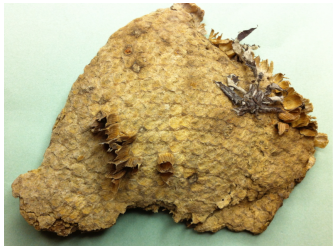

Eden-3

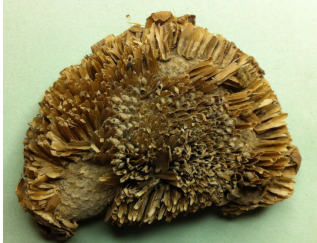

Eden-4

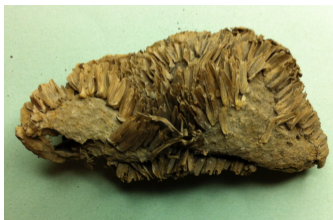

Eden-5

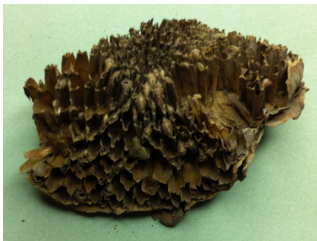

Eden-6

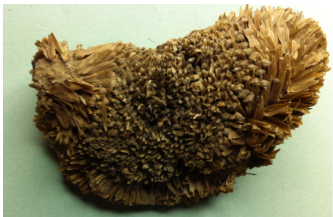

Eden-8

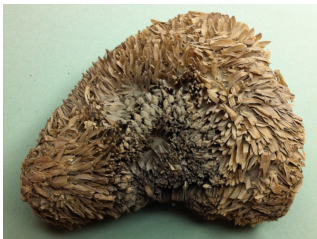

Eden-10

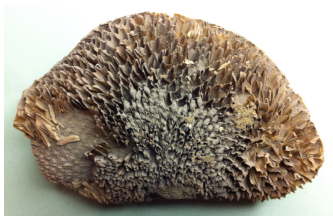

Eden-11

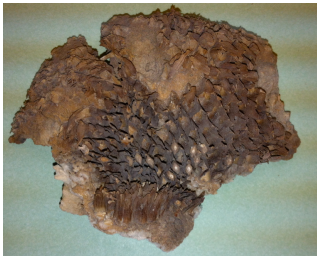

Eden-12

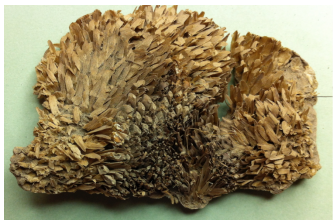

Eden-13

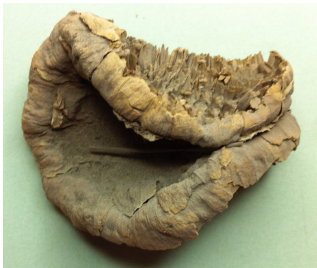

Eden-14

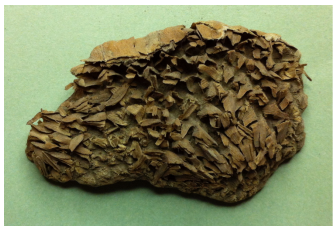

Eden-15

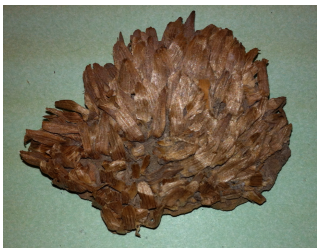

Eden-16

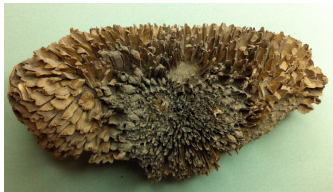

Supplement: Supplementary file 1 [file EVA-12-38-s001.pdf]

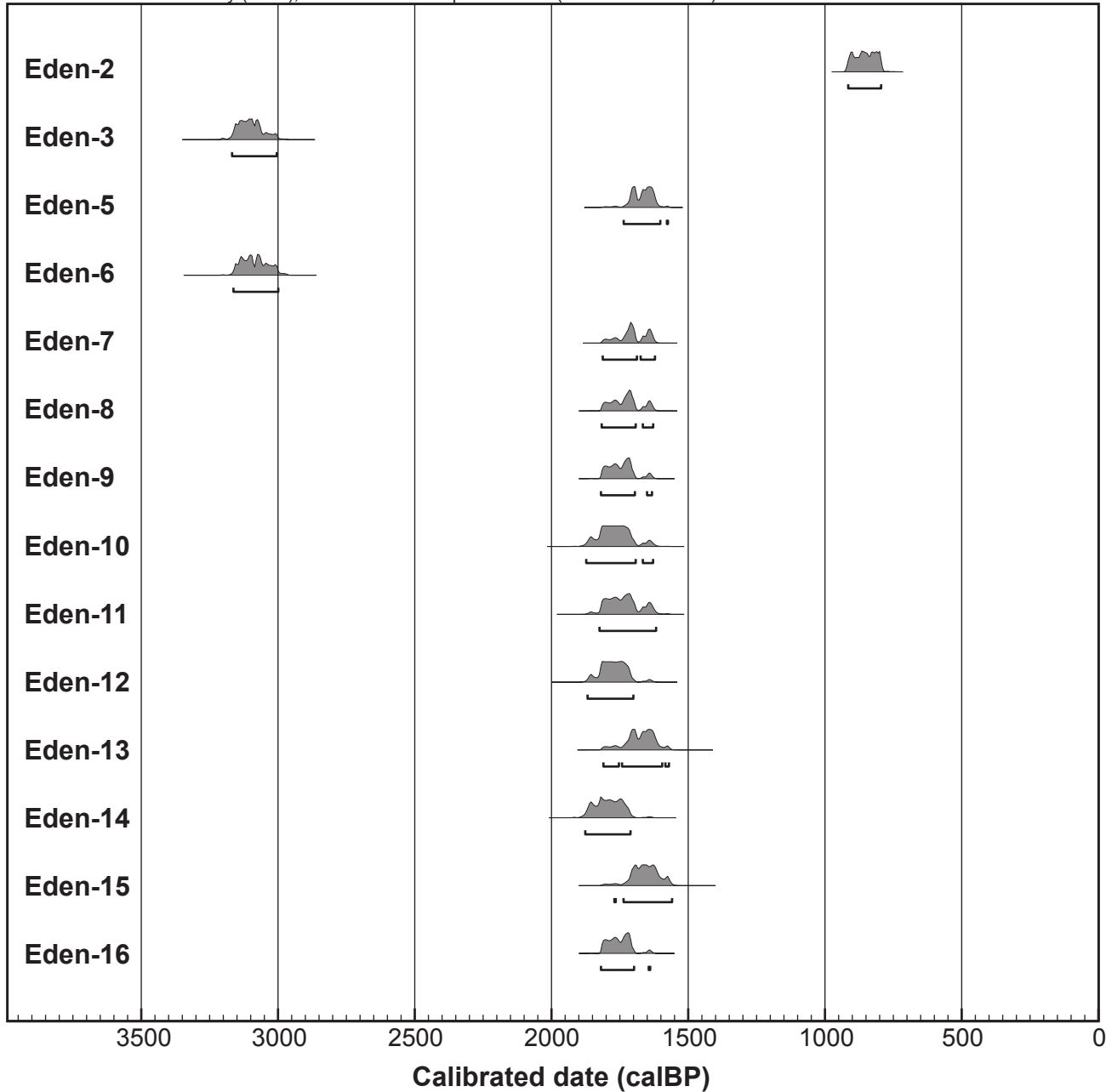

Supplement: Supplementary file 2 [file EVA-12-38-s002.pdf]

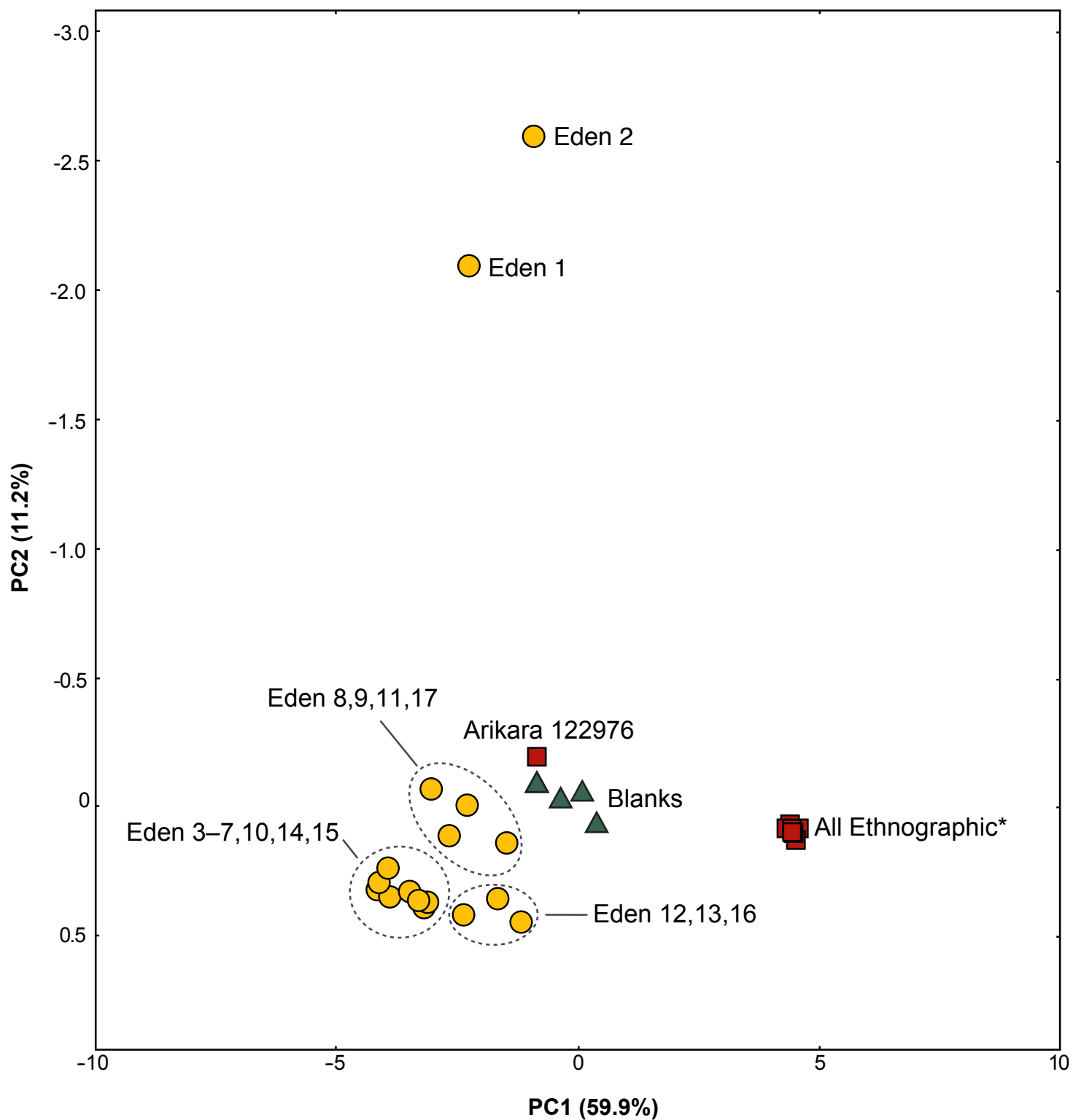

Supplement: Supplementary file 4 [file EVA-12-38-s004.pdf]
