# Supplementary material for: Ancient DNA reveals the timing and persistence of organellar genetic bottlenecks over 3,000 years of sunflower domestication and improvement
Source: Evol Appl. 2018 Feb 13;12(1):38–53. doi: 10.1111/eva.12594 (PMC6304678; doi:10.1111/eva.12594)
Supplement: Supplementary file 3 [file EVA-12-38-s003.pdf]

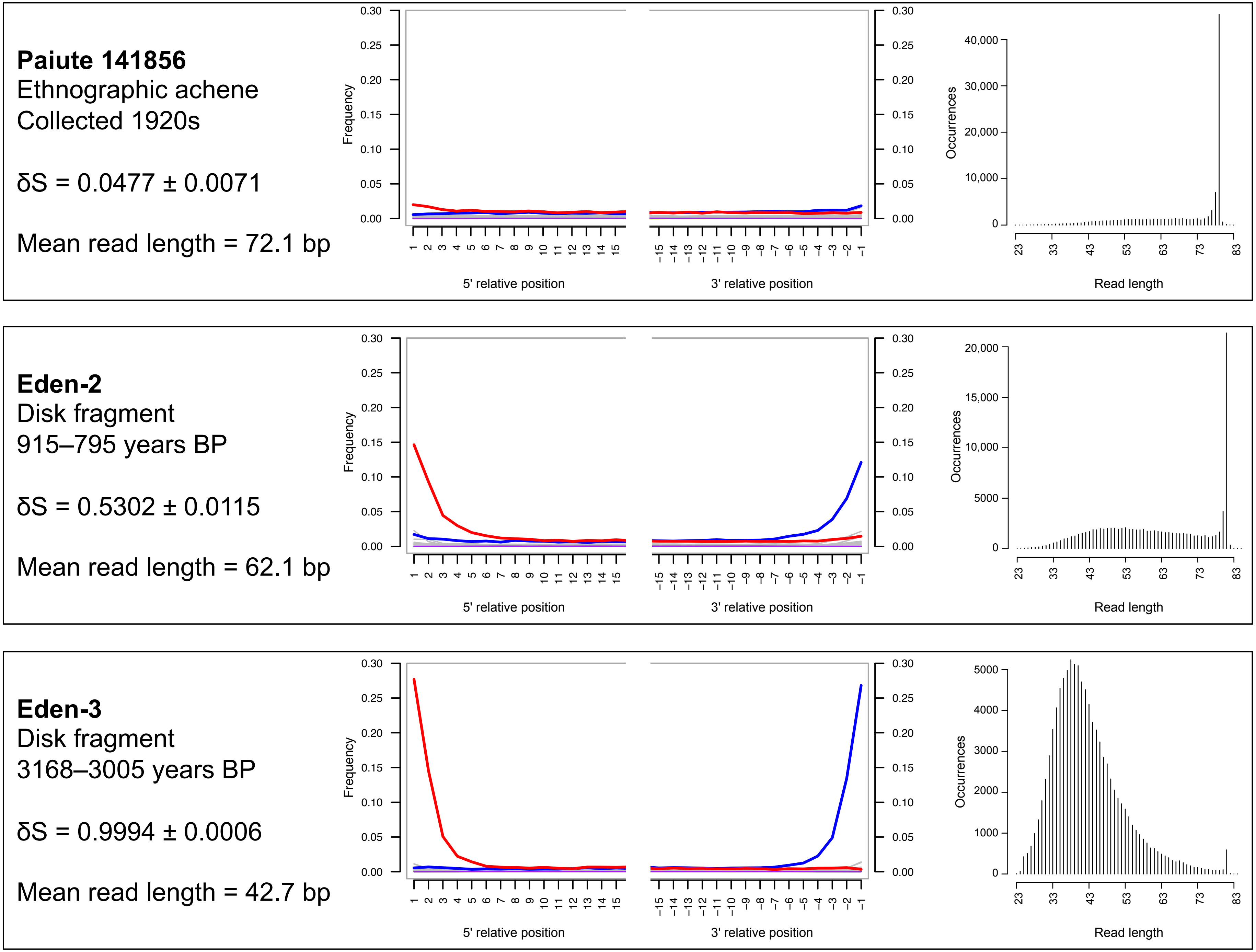
Paiute 141856
Ethnographic achene
Collected 1920s
δS = 0.0477 ± 0.0071
Mean read length = 72.1 bp
Eden-2
Disk fragment
915–795 years BP
δS = 0.5302 ± 0.0115
Mean read length = 62.1 bp
Eden-3
Disk fragment
3168–3005 years BP
δS = 0.9994 ± 0.0006
Mean read length = 42.7 bp
Frequency
5' relative position
3' relative position
Occurrences
Read length
